# Treatment of Mandibular Hypomobility by Injections into the Temporomandibular Joints: A Systematic Review of the Substances Used

**DOI:** 10.3390/jcm11092305

**Published:** 2022-04-20

**Authors:** Maciej Chęciński, Kamila Chęcińska, Zuzanna Nowak, Maciej Sikora, Dariusz Chlubek

**Affiliations:** 1 Department of Oral Surgery, Preventive Medicine Center, Komorowskiego 12, 30-106 Cracow, Poland; maciej@checinscy.pl; 2 Department of Glass Technology and Amorphous Coatings, Faculty of Materials Science and Ceramics, AGH University of Science and Technology, Mickiewicza 30, 30-059 Cracow, Poland; kamila@checinscy.pl; 3 Department of Temporomandibular Disorders, Medical University of Silesia in Katowice, Traugutta 2, 41-800 Zabrze, Poland; zuzannaewanowak33@gmail.com; 4 Department of Maxillofacial Surgery, Hospital of the Ministry of Interior, Wojska Polskiego 51, 25-375 Kielce, Poland; sikora-maciej@wp.pl; 5 Department of Biochemistry and Medical Chemistry, Pomeranian Medical University, Powstańców Wielkopolskich 72, 70-111 Szczecin, Poland

**Keywords:** temporomandibular joint, temporomandibular disorders, intra articular injection, viscosupplementation, platelet-rich plasma

## Abstract

Introduction: Hyaluronic acid, steroids and blood products are popularly injected into the temporomandibular joint (TMJs) to relieve pain and increase the extent of mandibular abduction. The purpose of this review is to identify other injectable substances and to evaluate them in the above-mentioned domains. Material and methods: The review included articles describing clinical trials of patients treated with intra-articular injections with or without arthrocentesis. Results: The following emerging substances were initially evaluated to be effective in treating TMJ pain and increasing the amplitude of mandibular abduction: analgesics, dextrose with lidocaine, adipose tissue, nucleated bone marrow cells and ozone gas. Discussion: Better effects of intra-articular administration are achieved by preceding the injection with arthrocentesis. Conclusions: The most promising substances appear to be bone marrow and adipose tissue.

## 1. Introduction

Paired temporomandibular joints (TMJs) are responsible for mandibular mobility. An open surgical access to TMJ is challenging due to anatomical conditions. The TMJ is located in the aesthetic preaural area, and access to it is difficult due to the course of the branches of the delicate facial nerve [1]. Neither of the open surgical approaches is ideal as they balance between sufficient insight and safety of anatomical structures [1,2,3].

For some TMJ interventions, such as fixing a joint prosthesis, treatment of advanced forms of ankylosis, or reposition and stabilization of intracapsular fractures, an open surgical approach is currently the only option [3]. Nevertheless, there are TMJ diseases that limit the extent of surgical cuts and preparations. Inspection of the joint area, removal of adhesions and polishing of the articular surfaces can be performed endoscopically from two small skin cuts [4]. A further reduction in invasiveness leads to the conversion of two cuts into two needle punctures, which allows for effective rinsing of the joint cavity [5]. As a result, the content of inflammatory mediators in the joint cavity is reduced and adhesions are removed. The use of only one injection needle is an extreme limitation of the invasiveness of surgical intervention within TMJ [5]. Such an intervention still allows various substances to be administered into the joint cavity and even to perform arthrocentesis [5,6].

Among the minimally invasive puncture techniques within TMJ, lavage of the joint cavity, supplementation of hyaluronic acid (HA) and administration of corticosteroids (CS) are commonly known and used [5,6,7]. TMJ arthrocentesis is effective in the domains of pain relief and increases the extent of mandibular abduction [8,9,10,11]. The administration of HA complements the main component of the synovial fluid and is also referred to as viscosupplementation [6]. Intra-articular administration of HA has been shown to be effective both as a stand-alone treatment and in combination with prior rinsing of the joint cavity [12,13,14]. The effectiveness of intra-articular steroid injection is uncertain [15]. There are many known complications of steroid administration, including edema, hypoaesthesia, skin hypopogmentation and even skin atrophy [16,17]. In recent years, injections of platelet-rich plasma (PRP) into the TMJ cavities have become popular and found to be effective [7,18,19,20,21,22]. Apart from autologous PRP, other self-derived blood products are also used: plasma rich in growth factors (PRGF) and injectable platelet-rich fibrin (I-PRF) [7,23]. Injecting analgesics, which are a non-homogeneous group of drugs with differently assessed effectiveness in this application, is also considered [24]. There are scarce reports, and no systematic reviews, on the administration of autologous transplants other than the patient’s blood to TMJs and of drugs other than those described above.

## 2. Aim

The aim of this review is to compile and evaluate comparative and efficacy-only studies on the administration of injectable substances into the cavities of the temporomandibular joints in the treatment of mandibular hypomobility and joint pain.

## 3. Materials and Methods

This review was based on the PRISMA guidelines and submitted for registration in the PROSPERO database [25,26]. The inclusion and exclusion criteria were established according to the PICOTS scheme (Table 1) [27].

The medical databases of EBSCO, Embase, Emcare, PubMed, SCOPUS and Web of Science, gray literature using a Google search engine and references were searched on 3 April 2022. The following search strategy was applied: “(temporomandibular OR tmj) AND (injection OR injections OR puncture OR punctures OR arthrocentesis OR lavage OR rinse OR rinsing OR viscosupplementation OR hyaluronic OR HA OR hyaluronan OR steroid OR steroids OR corticosteroid OR corticosteroids OR blood OR platelet OR PRP OR PGRF OR PRF OR I-PRF OR IPRF OR adipose OR marrow OR analgesic OR analgesics OR nsaid OR nsaids OR opioid OR opioids OR buprenorphine OR tenoxicam OR piroxicam OR tramadol OR fentanyl OR butorphanol OR chitosan OR morphine OR ozone) AND (clinical OR randomized) AND (trial OR rct)”. The reports have been selected blindly, and the data they contained were collected by two of the authors of the article (M.C. and K.C.). The screening and eligibility stages were carried out using the Rayyan tool (Qatar Computing Research Institute, Doha, Qatar and Rayyan Systems, Cambridge, MA, USA) [28]. The following data was extracted: (1) year of publication; (2) the name of the first author; (3) diagnosis; (4) type of intervention (administration or rinse and administration); (5) name of the substance administered; (6) average initial value of mandibular abduction for the study group, measured using the method adopted by the authors of the report; (7) final value of mandibular abduction, mean for the test group measured by the same method; (8) initial value of joint pain, mean for the study group, calculated by the authors of the report on the basis of the values for individual patients in accordance with the adopted study methodology; (9) the final value of joint pain, mean for the study group, calculated analogously to the initial value. The data was synthesized in tabular form. The effectiveness of treatment expressed as a change in the extent of mandibular abduction and reduction in joint pain was calculated by the authors of this review according to the formula

*e* = *f*/*i* × 100%, (1)

where *e* is the effectiveness resulting from the calculations for this study, *f* (7 or 9) is the final value given by the authors of the given report and *i* (6 or 8) is the initial value extracted from the same report. These calculations provided further data: (10) improvement in mandibular abduction; (11) reducing the value of joint pain [29,30,31,32]. In the case of mandibular mobility, values greater than 100% indicated good results of the therapy, and in the domain of pain, values less than 100% indicated a decrease in symptoms. Reports on hyaluronic acid, steroids and blood products were excluded from quantitative analysis due to the existence of the adequate systematic reviews mentioned in the introduction. The risk of bias for quantified trials was assessed by two authors (M.C. and K.C.) using the Revised Cochrane risk-of-bias tool for randomized trials, as all the studies were randomized trials. [33]. The analyses (including regression analysis) and graphic presentation of the data were performed with the use of Google office software (Google LLC, Mountain View, CA, USA).

## 4. Results

**Figure 1:**
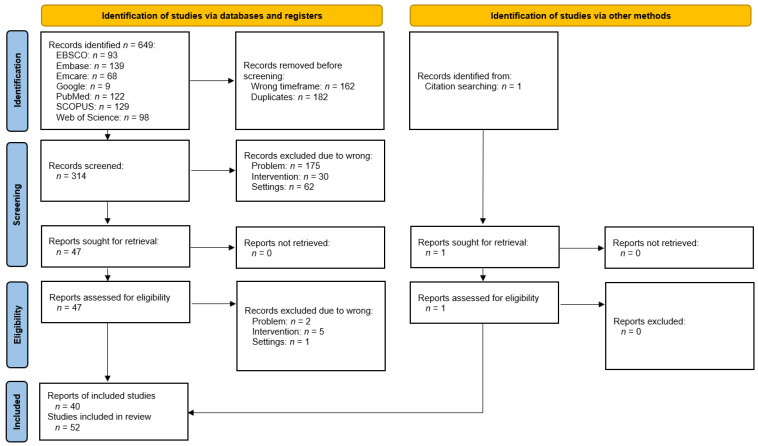
Studies selection process.

All medical database searches performed gave a total of 649 records (Figure 1). Of these, 162 out-of-date entries were automatically deleted and 182 duplicates were manually removed. 305 records have been qualified for blind screening by two authors. At this stage, 267 reports were rejected, most of them relating to the wrong group of patients, including wrong diagnoses or non-human studies. Review papers and case reports were also discarded at this stage. Authors’ compliance at the screening phase was 98.5% (Cohen’s k: 0.89). A search of websites and references yielded another 10 results suitable for full-text analysis. Full content of all proceeded reports was acquired. At the stage of eligibility, eight papers listed in Table 2 were rejected. Thus, 40 reports containing 52 studies meeting the assumed criteria for systematic review were qualified for synthesis (Table 3). The study of injectables other than HA, CS and blood products was assessed for the risk of bias as shown in Table 4.

**Figure 2:**
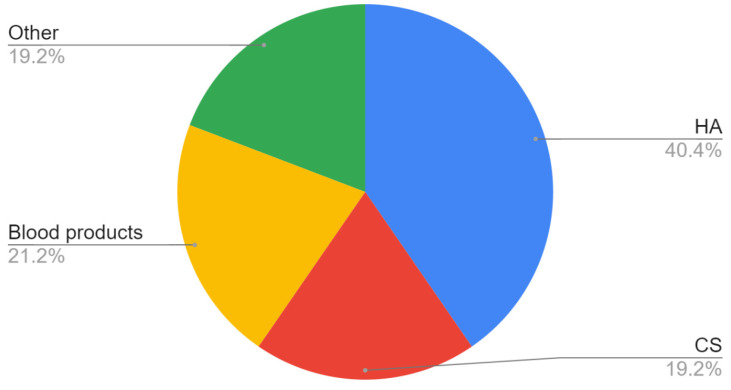
Use of individual substances: HA—hyaluronic acid; CS—corticosteroids.

**Figure 3:**
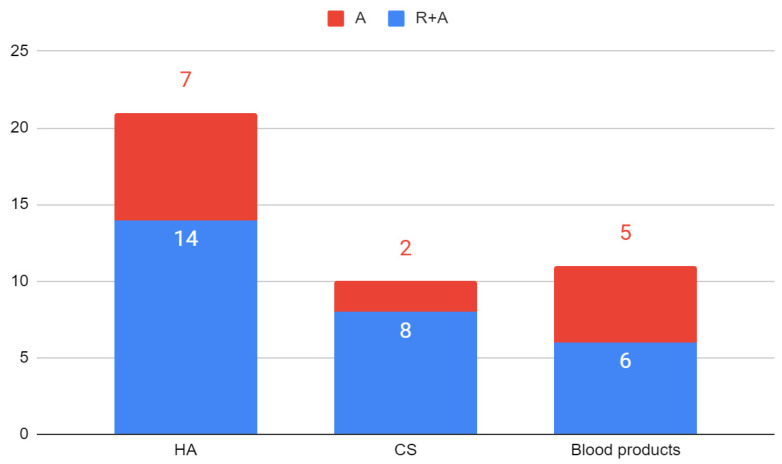
Number of studies with and without prior arthrocentesis (three most popular substances): A—administration; R—rinse; HA—hyaluronic acid; CS—corticosteroids.

In line with the assumptions of the review, a total of 15 substances and combinations of substances injected into the cavities of the temporomandibular joints were identified. The most commonly studied over the past 10 years have been HA (40.4%), CS (19.2%), and blood products (21.2%) with or without prior arthrocentesis (Figure 2 and Figure 3). In one study a combination of HA and CS reduced TMJ pain in 91% and increased mandibular abduction in 60% of patients who initially reported these complaints. [75]. Among blood products, PRP is the most commonly used (15.4% of all substances).

**Figure 4:**
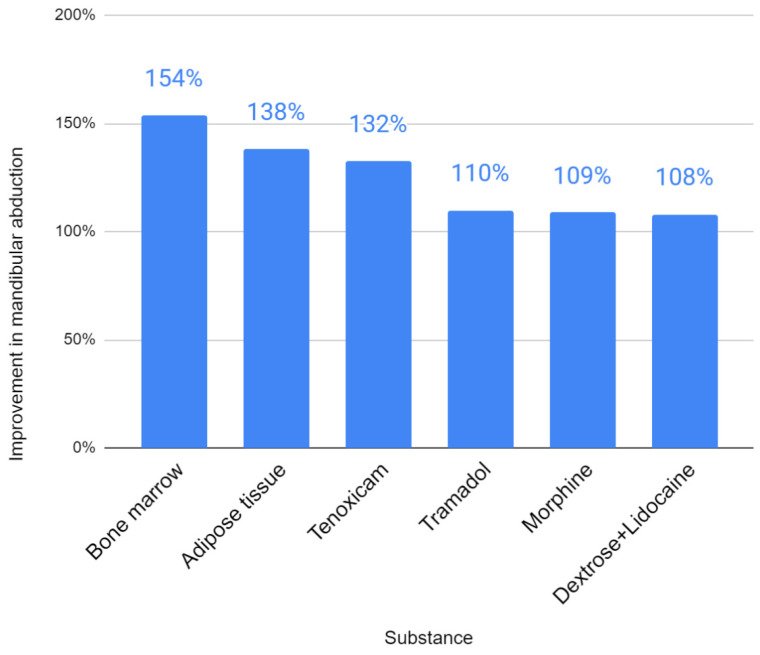
Improvement in mandibular abduction (the greater the value, the better the result).

**Figure 5:**
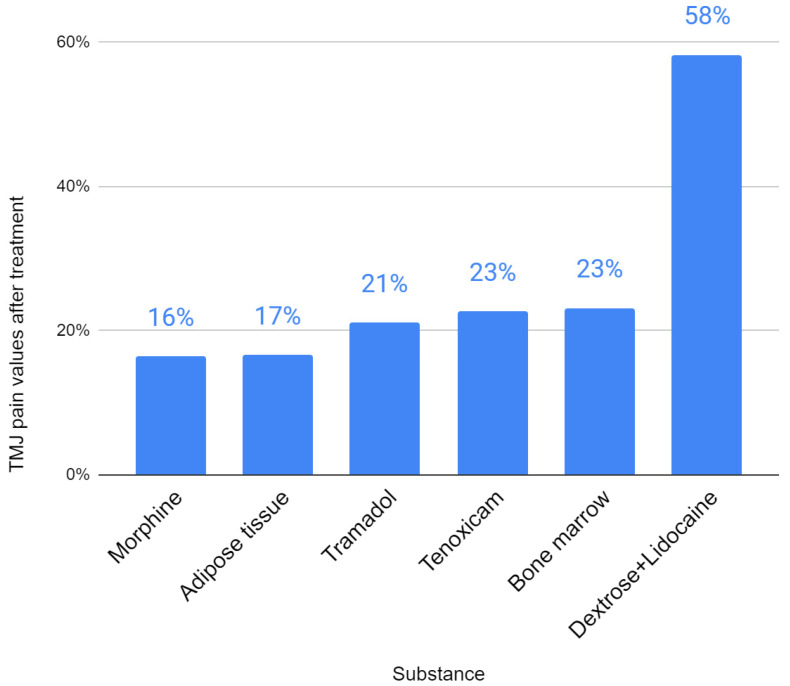
Final TMJ pain values after treatment with individual injectables expressed as a percentage (the pain value before treatment was 100% in each case).

The conducted review allowed for the identification of other, less popularly tested injectables, such as autogenous transplants, monosaccharide in combination with an anesthetic, analgesics and gas (Table 5). With regard to the effect on the extent of mandibular abduction, the bone marrow showed the greatest efficacy (154%) of the rarely used substances (Figure 4). The action of dextrose with lidocaine, morphine and tramadol did not increase the mobility of the mandible by more than 15%. The results of mandibular lateral mobility and protrusive mobility have not been reported for any of these substances. Baseline pain, defined as 100% for the purposes of the analysis, significantly decreased in each of the studies (Figure 5). Strong pain-reducing effect was achieved by analgesics and autografts: morphine (16% of initial complaints), adipose tissue (17%), tramadol (21%), bone marrow (23%) and tenoxicam (23%). Dextrose with lidocaine gave very divergent results in different studies (from 33% to 76% of initial pain). It was not possible to evaluate ozone gas in any of the two domains due to different outcome measures.

**Figure 6:**
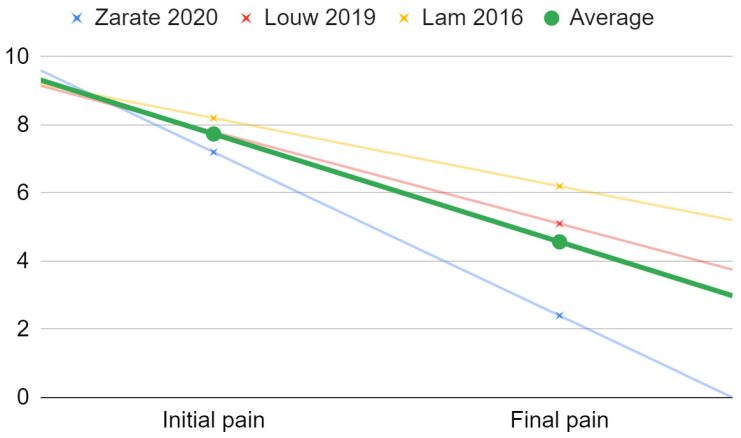
Linear regression model of pain intensity in dextrose and lidocaine therapy.

Among the substances other than those already assessed in the previously published meta-analyzes, only the administration of dextrose with lidocaine was documented in more than one report, which limited the possibility of the meta-analysis to this one substance [12,18,20,21,24,44,49,62]. The amplitude of mandibular abduction was reported only in two of the three reports, which precludes any statistical analysis. The three initial and three final pain values obtained from the study allowed for fitting a linear regression model of pain intensity of the formula −3.2x + 7.7 with standard deviations of 0.5 and 2.0 for the initial and final TMJ pain intensity, respectively (Figure 6).

## 5. Discussion

### 5.1. Hyaluronic Acid

In primary studies indexed as clinical trials in the last 10 years, HA injections dominate. This substance is either used alone or administered after arthrocentesis. Both of these methods result in an increase in the mobility of the mandible [6,7,78]. The intra-articular administration of HA was the only procedure used in the following diagnoses: internal derangement, disk displacement with reduction, degenerative disorders Administration of HA associated with arthrocentesis was used in all the above indications and additionally in the treatment of osteoarthritis, disk displacement without reduction and unspecified joint pain [47,52,58]. The current systematic review of the efficacy of intra-articular hyaluronic acid in the treatment of reduced mobility and pain in TMJ suggests that the second and subsequent administrations of the drug are less effective than the first [12].

### 5.2. Corticosteroids

Arthrocentesis combined with CS administration was effective in increasing mouth opening range in the following diagnoses: internal derangement, osteoarthritis, disk displacement without reduction, degenerative disorders, and unspecified joint pain [43,52,56,58,68,73]. The fact that arthrocentesis with CS administration increases the mobility of the mandible, may however be the result of the joint lavage itself [7,48]. A single study involving the administration of CS alone did not show any significant increase in the extent of mandibular abduction [48]. It was observed in a group of patients with a common feature of joint pain diagnosis [48]. On the other hand, preceding the administration of CS with arthrocentesis is effective in the analyzed domain [43,52,56,58,68,73]. It cannot be ruled out that the improvement in the mouth opening occurs due to the benefits of rinsing of the joint cavity, not from the drug administration [7,48]. However, this issue requires separate research.

### 5.3. Blood Products

Among the various blood products used in medicine, PRP, I-PRF and PRGF have been identified for injection into TMJs [40,76,77,78,79,80,81,82,83]. The effectiveness of blood products results, among others, from the content of platelets, cytokines and growth factors, which are successfully used in supporting wound healing, among others in dentistry [81,82,83,84,85]. PRP is used both alone and in combination with arthrocentesis [60,63,64,67,80]. Both approaches are known to be beneficial in terms of increasing the mobility of the mandible [7,19,21,86]. In the material collected for the review, osteoarthritis was treated in both ways [60,63,64]. PRP administration as the only procedure was effective in terms of increasing mandibular abduction amplitude in the diagnoses of disk displacement with reduction and not specified joint pain. I-PRF was used only after arthrocentesis, and PRGF was used without rinsing the joint [38,40,79]. Data on the use of the latter substance are derived from only one report, describing a study without a control group [79].

### 5.4. Analgesics

In the course of the literature search, it was found that the TMJs cavities are therapeutically administered with morphine, tramadol, tenoxicam and lidocaine as an additive to dextrose [44,49,52,66,87]. A systematic review of the effectiveness of intra-articular analgesics by Liu et al., in 2021 showed divergent results for the NSAIDs and opioids [24]. These authors noted the lack of statistical significance in relation to the control groups in the results of NSAID treatment, which questioned the effectiveness of the administration of these drugs [24]. Compared to opioids, in the course of the analysis in this review, tenoxicam gave the final results of mandibular mobilization not much worse than tramadol and an approximately four-fold decrease in pain, similar to tramadol [52,66]. This effect may be largely attributed to prior arthrocentesis [24,66]. For opioids, there are likely to be statistically significant differences between the groups treated with drugs in combination with arthrocentesis and the joint lavage alone [24,87,88,89,90,91].

### 5.5. Dextrose

Dextrose solution is administered intra-articularly with the addition of lidocaine, which is referred to as prolotherapy. In the study by Zarate et al., a decrease in pain symptoms was shown to the level of 33% of the initial value, which, however, was not confirmed in the other two reports (76–65%) [44,49,62]. The increase in mandibular mobility did not exceed 12% in the analyzed studies [44,49]. The obtained results are clearly worse than in the case of administering analgesics or transplants, which perhaps should be explained by the lack of arthrocentesis before the prolotherapy [39,44,45,49,62]. Sit et al., indicate that a review of studies on dextrose injection shows statistically significant results in favor of prolotherapy in relation to the control groups [92].

### 5.6. Transplants

Self-derived transplants constitute a non-homogeneous group of injectables. These include, first of all, the blood products already discussed. Apart from them, there are the first experimental and clinical studies on intra-articular administration of adipose tissue and bone marrow cells [39,45,93,94,95]. The promising results of these therapies do not exempt them from caution in their implementation [39,45].

### 5.7. Ozone Gas

Ozone at the tissue level is anti-inflammatory and stimulates the immune system [71,96,97,98]. The research conducted so far on ozone administration into TMJs cavities is insufficient to draw conclusions on this subject [97]. The Daif et al. study analyzed in this systematic review cannot be compared with other therapies due to different outcome measures [71].

### 5.8. Differential Diagnosis

Apart from intra-articular injections, physiotherapy, pharmacotherapy, splint therapy and injections into the masticatory muscles are also used in the treatment of temporomandibular joint dysfunctions [6,99,100,101]. The latter are applicable when it is possible to diagnose that the pain and movement restrictions are of muscle origin, not articular [6,99]. The limitation of the mobility of the mandible may also result from a mechanical obstruction, including trauma, various stages of ankylosis of the temporomandibular joint (mainly traumatic) and hyperplasia of the coronoid processes [3,102,103,104]. A thorough subjective and physical examination as well as three-dimensional imaging of the temporomandibular joints can therefore prevent implementation of an inadequate therapy.

### 5.9. Limitations

The limitation of this review is the difficulty in formulating a strategy to search for substances whose names we want to identify, which may have resulted in the overlooking of other injectables. Therefore it seems justified to undertake further reviews aimed at individual identified substances.

## 6. Conclusions

52 studies on injection into the cavities of the temporomandibular joints in 40 reports compliant with the adopted systematic review criteria were identified. Intra-articular administrations of hyaluronic acid (40.4%), corticosteroids (19.2%) and blood products (21.2%) dominated. Emerging methods of treatment of mandibular hypomobility are intra-articular injections of analgesics, dextrose, self-derived transplants and ozone gas (17.3% in total). The most promising substances are self-derived transplants: bone marrow and adipose tissue. Among these substances, better results in mandibular mobility and reduction in joint pain have been achieved with therapies including pre-injection arthrocentesis.

## Figures and Tables

**Table 1:** Criteria for including and excluding studies from the review.

	Inclusion Criteria	Exclusion Criteria
Patient description	Temporomandibular joint (TMJ) disease	Animal studies
Intervention description	TMJ injection with or without arthrocentesis	TMJ injection as part of a more complex treatment; any additional intervention
Comparators description	Placebo or other injectable group with a similar size (+/−10%) and assessed for the same outcomes as the study group or no control group	None
Outcomes description	Primary outcome: (1) improvement of mandibular abduction; secondary outcomes: (2) improvement of mandibular lateral mobility, (3) improvement of mandibular protrusion, (4) pain relief of TMJ	None
Timeline	Papers published from 1 January 2012 to 3 April 2022	
Settings	Clinical trials	No abstract available

**Table 2:** Records excluded at the eligibility stage.

Report	PICOS Criterion	Reason for Exclusion
Cömert Kılıç, S. Does glucosamine, chondroitin sulfate, and methylsulfonylmethane supplementation improve the outcome of temporomandibular joint osteoarthritis management with arthrocentesis plus intra-articular hyaluronic acid injection. A randomized clinical trial. *J*. *Craniomaxillofac*. *Surg*. **2021**, *49*, 711–718.	Intervention	Oral administration
Haghighat, S.; Oshaghi, S. Effectiveness of Ozone Injection Therapy in Temporomandibular Disorders. *Adv*. *Biomed*. *Res*. **2020**, *28*, 73.	Settings	Review article
Sakalys, D.; Dvylys, D.; Simuntis, R,.; Leketas, M. Comparison of Different Intraarticular Injection Substances Followed by Temporomandibular Joint Arthroscopy. *J*. *Craniofac*. *Surg*. **2020**, *31*, 637–641.	Intervention	Additional intervention
Özkan, H.S.; Irkören, S.; Karaca, H.; Yıldırım, T.D.; Çiçek, K.; Tataroğlu, C. Effects of Intra-Articular Platelet-Rich Plasma Administration in Temporomandibular Joint Arthritis: An Experimental Study. *Meandros* *Med*. *Dent*. *J*. **2018**, *19*, 198–204	Patient	Animal studies
Buendía-López, D.; Medina-Quirós, M.; Fernández-Villacañas Marín, M.Á. Clinical and radiographic comparison of a single LP-PRP injection, a single hyaluronic acid injection and daily NSAID administration with a 52-week follow-up: a randomized controlled trial. *J*. *Orthop*. *Traumatol*. **2018**, *19*, 3.	Patient	Wrong joint
Campbell, B.K.; Fillingim, R.B.; Lee, S.; Brao, R.; Price, D.D.; Neubert, J.K. Effects of High-Dose Capsaicin on TMD Subjects: A Randomized Clinical Study. *JDR* *Clin*. *Trans*. *Res*. **2017**, *2*, 58–65.	Intervention	Transdermal administration
Baker, Z.; Eriksson, L.; Englesson Sahlström, L.; Ekberg, E. Questionable effect of lavage for treatment of painful jaw movements at disc displacement without reduction: a 3-year randomised controlled follow-up. *J*. *Oral*. *Rehabil*. **2015**, *42*, 742–750.	Intervention	Extra-articular administration
Sahlström, L.E.; Ekberg, E.C.; List, T.; Petersson, A.; Eriksson, L. Lavage treatment of painful jaw movements at disc displacement without reduction. A randomized controlled trial in a short-term perspective. *Int*. *J*. *Oral* *Maxillofac* *Surg*. **2013**, *42*, 356–363.	Intervention	Extra-articular administration

**Table 3:** Results. ID—internal derangement [34,35]; P—TMJ pain according to ICOP [34,36]; OA—osteoarthritis [34,37]; DDwR—disk displacement with reduction [34,37]; DDworR—disk displacement without reduction [34,37]; DD—degenerative disorders [34,37]; R—rinse; A—administration; HA—hyaluronic acid; CS—corticosteroids; PRP—platelet rich plasma; I-PRF—injectable platelet rich fibrin; PRGF—plasma rich in growth factors *—randomized controlled trial.

Section 1: Comparative Studies					
Publication Year	First Author	Diagnosis	Intervention	Substance	Comparison Group
2022	Ghoneim [38]	DDwR	R+A	I-PRF	R *
2021	Sembronio [39]	ID, OA	R+A	Adipose tissue	R+HA *
2021	Sembronio [39]	ID, OA	R+A	HA	R+Adipose tissue *
2021	Karadayi [40]	ID	R+A	I-PRF	R *
2021	Jacob [41]	DDwR, DDwoR	R+A	PRP	R *
2021	Jacob [41]	DDwR, DDwoR	R+A	HA	R *
2021	Singh [42]	ID	R+A	PRP	R *
2020	Dolwick [43]	P	R+A	CS	R+Placebo *
2020	Zarate [44]	P	A	Dextrose+Lidocaine	Lidocaine *
2019	De Riu [45]	DD	R+A	HA	R+Bone marrow *
2019	De Riu [45]	DD	R+A	Bone marrow	R+HA *
2019	Yilmaz [46]	ID	A	HA	R+HA *
2019	Yilmaz [46]	ID	R+A	HA	HA *
2019	Bergstrand [47]	OA	R+A	HA	R *
2019	Isacsson [48]	P	A	CS	Placebo *
2019	Louw [49]	P	A	Dextrose+Lidocaine	Lidocaine *
2019	Gokçe Kutuk [50]	P	A	HA	CS *
2019	Gokçe Kutuk [50]	P	A	CS	HA *
2019	Gokçe Kutuk [50]	P	A	PRP	CS *
2019	Diaz [51]	P	R+A	CS	R+Placebo *
2018	Yapici-Yavuz [52]	DDwoR	R+A	CS	R *
2018	Yapici-Yavuz [52]	DDwoR	R+A	HA	R *
2018	Yapici-Yavuz [52]	DDwoR	R+A	Tenoxicam	R *
2017	Ozdamar [53]	ID	R+A	HA	R *
2017	Gorrela [54]	DDwR, DDwoR	R+A	HA	R *
2017	Gurung [55]	OA	R+A	HA	R *
2016	Cömert Kiliç [56]	OA	R+A	CS	R *
2016	Patel [57]	ID	R+A	HA	R *
2016	Bouloux [58,59]	P	R+A	CS	R *
2016	Bouloux [58,59]	P	R+A	HA	R *
2016	Cömert Kiliç [60]	OA	R+A	PRP	R+HA *
2016	Korkmaz [61]	DDwR	A	HA	Splint therapy *
2016	Lam [62]	P	A	Dextrose+Lidocaine	Lidocaine *
2015	Cömert Kiliç [63]	OA	R+A	PRP	R *
2015	Hegab [64]	OA	A	HA	PRP *
2015	Hegab [64]	OA	A	PRP	HA *
2015	Guarda-Nardini [65]	DD	A	HA	R+HA *
2015	Sipahi [66]	ID	R+A	Morphine	R+Placebo *
2015	Sipahi [66]	ID	R+A	Tramadol	R+Placebo *
2014	Hancı [67]	DDwR	A	PRP	R *
2014	Tabrizi [68]	ID	R+A	CS	R *
2013	Bustaman [69]	OA	A	HA	Placebo *
2012	Guarda-Nardini [70]	DD	R+A	HA	HA*
2012	Daif [71]	ID	A	Ozone gas	Oral drugs *
2012	Guarda-Nardini [72]	DD	R+A	HA	HA *
2012	Manfredini [73]	DD	R+A	CS	R *
2012	Manfredini [73]	DD	R+A	HA	R *
2012	Huddleston Slater [74]	P	R+A	CS	R *
Section 2: before-and-after studies					
Publication	First author	Diagnosis	Intervention	Substance	
2020	Singh [75]	OA	A	CS+HA	
2020	Sikora [6]	P	A	HA	
2019	Giacomello [76]	OA	A	PRGF	
2014	Pihut [77]	P	A	PRP	

**Table 4:** Risk of bias assessment: Domain 1—Risk of bias arising from the randomization process; Domain 2—Risk of bias due to deviations from the intended interventions; Domain 3—Missing outcome data; Domain 4—Risk of bias in measurement of the outcome; Domain 5—Risk of bias in selection of the reported result; Overall—Overall risk of bias.

First Author	Domain 1	Domain 2	Domain 3	Domain 4	Domain 5	Overall
Sembronio [39]	Low	Moderate	Low	Low	Low	Moderate
Zarate [44]	Low	Low	Low	Low	Low	Low
De Riu [45]	Low	Moderate	Low	Low	Low	Moderate
Louw [49]	Low	Low	Low	Low	Low	Low
Yapici-Yavuz [52]	Low	Moderate	Low	Low	Low	Moderate
Lam [62]	Low	Low	Low	Low	Low	Low
Daif [71]	Low	Moderate	Low	Low	Low	Moderate

**Table 5:** Quantitative analysis.

First Author	Substance	Initial Abduction	Final Abduction	Initial Pain	Final Pain	Abduction Improvement	Pain Improvement
Sembronio [39]	Adipose tissue	30.7	42.4	7.2	1.2	138%	17%
Zarate [44]	Dextrose+Lidocaine	38.7	43.4	7.2	2.4	112%	33%
De Riu [45]	Bone marrow	22	33.8	8.2	1.9	154%	23%
Louw [49]	Dextrose+Lidocaine	43.4	45	7.8	5.1	104%	65%
Yapici-Yavuz [52]	Tenoxicam	25.3	33.5	7.5	1.7	132%	23%
Lam [62]	Dextrose+Lidocaine			8.2	6.2		76%
Sipahi [66]	Morphine	37.7	41	7.3	1.2	109%	16%
Sipahi [66]	Tramadol	34.6	38	7.1	1.5	110%	21%
Daif [71]	Ozone gas	No data	No data	No data	No data	No data	No data

## Data Availability

Availability of data, code and other materials: The protocol of the systematic review is available in the PROSPERO database under the number CRD42022318742. The entirety of the collected data is presented in the content of this article.
